# Attenuation artifacts in light sheet fluorescence microscopy corrected by OPTiSPIM

**DOI:** 10.1038/s41377-018-0068-z

**Published:** 2018-10-03

**Authors:** Jürgen Mayer, Alexandre Robert-Moreno, James Sharpe, Jim Swoger

**Affiliations:** 1 Centre for Genomic Regulation (CRG), The Barcelona Institute of Science and Technology, Dr. Aiguader 88, 08003 Barcelona, Spain; 2 Universitat Pompeu Fabra (UPF), Barcelona, Spain; 3 Institució Catalana de Recerca i Estudis Avançats (ICREA), Pg. Lluis Companys 23, 08010 Barcelona, Spain; 4 Present Address: European Molecular Biology Laboratory (EMBL), Dr. Aiguader 88, 08003 Barcelona, Spain

## Abstract

Light sheet fluorescence microscopy (LSFM) is rapidly becoming an essential technology for mesoscopic imaging of samples such as embryos and adult mouse organs. However, LSFM can suffer from optical artifacts for which there is no intrinsic solution. The attenuation of light due to absorbing material causes “shadow” artifacts along both the illumination and detection paths. Several approaches have been introduced to reduce this problem, including scanning illumination and multi-view imaging. However, neither of these approaches completely eliminates the problem. If the distribution of the absorbing material is complex, shadows cannot be avoided. We introduce a new approach that relies on multi-modal integration of two very different mesoscopic techniques. Unlike LSFM, optical projection tomography (OPT) can operate in transmission mode to create a voxel map of the 3D distribution of the sample’s optical attenuation. Here, we demonstrate a hybrid instrument (OPTiSPIM) that can quantify this attenuation and use the information to correct the shadow artifacts of LSFM.

## Imaging: Biological imaging technique comes out from the shadows

Scientists in Spain have developed a new method that combines two different techniques for improving the quality of images produced by light sheet fluorescence microscopy (LSFM), a tool that has revolutionized the imaging of biological samples. Although LSFM is a becoming an increasingly essential tool in biological imaging, it suffers from ‘shadows’ or ‘stripe artifacts’ when samples contain regions that significantly attenuate light, such as eye pigmentation. Now, Jim Swoger and colleagues from the Center for Genomic Regulation in Barcelona have developed a technique that measures the attenuation and uses three-dimensional mapping to correct for artifacts. By combining Optical Projection Tomography and multi-view LSFM imaging modalities into a single hybrid system, called OPTiSPIM, the researchers have developed a method that produces more accurate images of biological materials, such as mice and rodent embryos and organs.

## Introduction

In recent years, techniques for imaging three-dimensional (3D) mesoscopic samples—those ranging in size from tens of microns to more than a centimeter—have emerged to fill a previously unoccupied niche in the field of biological imaging. Both traditional microscopy^1^ and recently developed “nanoscopy” methods^2–4^ are well suited to single cells or small groups of cells but are not optimal for imaging larger samples, such as fly, fish, and mammalian embryos, or intact organs of adult model systems such as the mouse brain, lung, or pancreas. At the other end of the scale, macroscopic imaging methods, such as magnetic resonance imaging (the abbreviations and symbols used in this paper are summarized in Supplementary Table 2), are well suited for whole organisms ranging from rats to humans; however, they have lower a resolution and reduced power to visualize specific molecular labels.

One of the first mesoscopic techniques to fill this “imaging gap” was optical projection tomography (OPT)^5^—an optical implementation of computed tomography that is analogous to X-ray computed tomography. OPT collects a series of projection images of the sample from different angles and computationally reconstructs a 3D image of the sample using filtered back-projection or algebraic reconstruction techniques^6^. OPT has been implemented for both fixed tissue^7–9^ and live imaging^10–13^, and for applications such as developmental biology^14^, diabetes studies^8^, and immunology^15^. One of the advantages of OPT is that it can be used for fluorescent (fluorescent proteins and fluorophore-labeled antibodies) and non-fluorescent (natural pigmentations and colored dyes) contrasts.

Another important imaging technique that is suitable for mesoscopic samples is light sheet fluorescence microscopy (LSFM)^16^, which includes implementations such as orthogonal-plane fluorescence optical sectioning^17^, selective plane illumination microscopy (SPIM)^18^, ultramicroscopy^19^, and digital scanned laser LSFM (DSLM)^20^. The common theme of these techniques is the excitation of fluorescence by a thin sheet of light that is perpendicular to the detection axis and coincides with the focal plane of a wide-field microscope, thus allowing fluorescence imaging with intrinsic optical sectioning, minimal photo-bleaching/photo-damage, the use of relatively low numerical aperture objective lenses with long working distances.

Within the mesoscopic realm, LSFM systems have been designed primarily for two types of samples: relatively small, transparent objects that can be imaged live and larger or more opaque samples that require fixation and chemical clearing for 3D imaging. Examples of the former include studies on the development of fruit flies^21–23^ and zebrafish^20,24^ and the neuronal activity in intact zebrafish^25^ and mice^26^. The use of LSFM for larger, fixed samples has been very appealing for neurologists wishing to understand the complex structure and function of the brain^19,27^. However, it has also become valuable for many other samples, including studies of the inner ear^28^, immunology^29,30^, and multi-cellular tumor spheroids^31^.

**Fig. 1:**
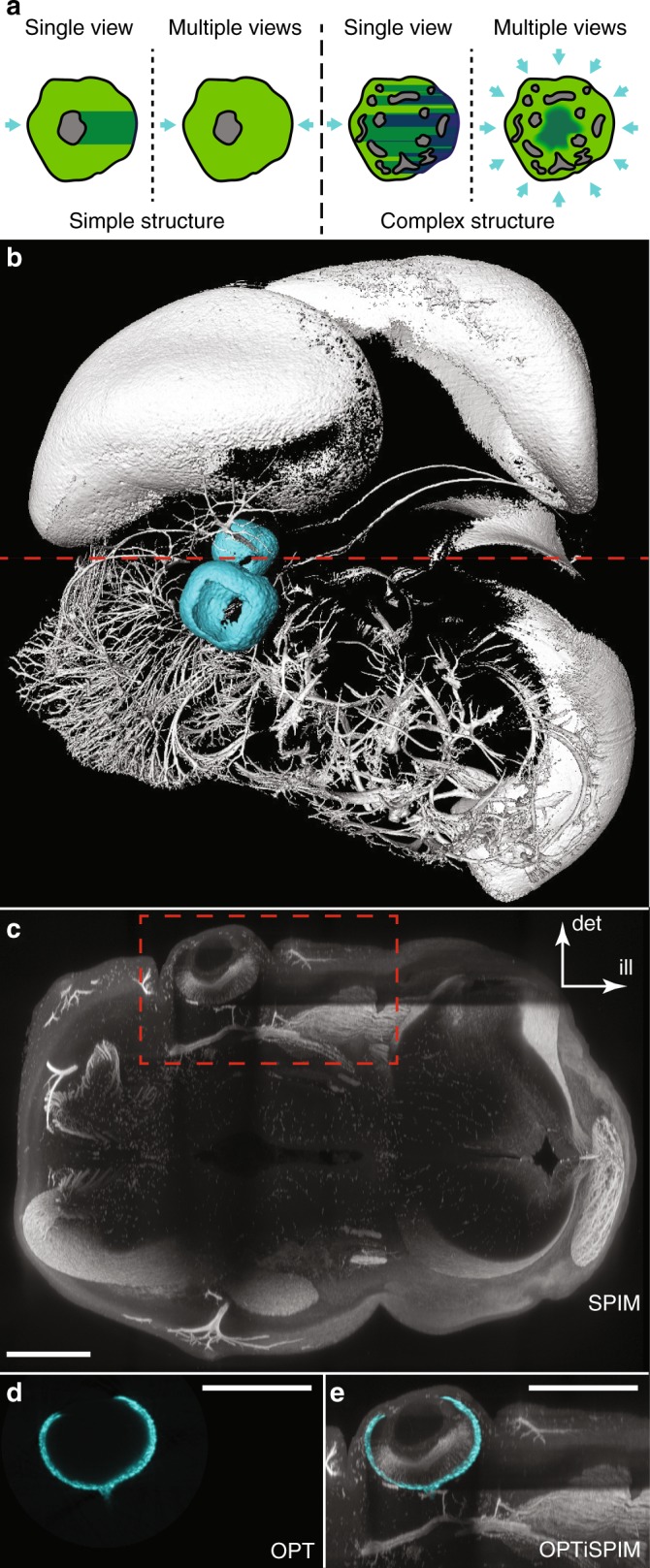
Absorption artifacts in light sheet imaging. **a** Attenuation artifacts in simple vs. complex structures. Green: fluorescent regions; gray: attenuating regions; cyan arrows: illumination directions (for simplicity, the effects of attenuation on the emitted fluorescence are not shown); dark green: non/poorly illuminated regions. Left: When the attenuating region is relatively simple (geometrically), the artifacts can be corrected by multi-view reconstruction (in the case shown, two views are sufficient). Right: For more complex attenuating structures, there are generally regions in the sample that are not clearly illuminated by any view and thus are not properly corrected by standard multi-view reconstructions. **b** Surface rendering of a cleared embryonic stage E12.5 mouse head, immunolabeled for Tuj1 (class III β-tubulin, a neuronal marker). The sample was imaged using both LSFM (Tuj1, white surface) and tOPT (eye pigments, cyan surface). The retina contains pigmented cells that significantly absorb light and, therefore, create contrast to visualize the eyeball. Although absorption artifacts are present in the LSFM image, if they are not recognized for what they are, they may be misinterpreted as an intrinsically weaker signal. **c** A 130-µm-thick slice through the fluorescence image at the level of the red dashed line in **b**. The light-absorbing retina casts two shadows: the horizontal shadow on the right indicates where illumination (from the left) was considerably reduced, and the vertical shadow (below the eye) indicates the regions obscured from the view of the objective lens used for detection, which is above (ill = illumination, det = detection). **d**, **e** show the reconstruction of the eye pigmentation from a tOPT scan in the region indicated by the red box in (**c**) and the overlay of the pigmentation and the fluorescence signal imaged in the SPIM mode, respectively. The shadow artifacts in the fluorescence data are well aligned with the eye pigmentation. Scale bars: 500 µm

LSFM is rapidly gaining in popularity due its clear advantages for imaging thick samples; however, “shadows” or “stripe artifacts” occur when the sample contains regions that significantly attenuate light (such as the eye pigmentation in Fig. 1 or the nitro blue tetrazolium/5-bromo-4-chloro-3-indolyl phosphate (NBT/BCIP) staining in Supplementary Fig. 3). This attenuation may affect both the excitation light sheet before it reaches the fluorophores (this effect is visible as the dark shadow to the right of the eye in Fig. 1c) and the emitted fluorescence before it reaches the camera (the reduced signal below the eye in Fig. 1c). These artifacts can cause serious problems for quantitative data analysis or even undermine the ability to clearly see certain structures^28,32,33^, for example, the shadows in Fig. 1c would make accurate mapping of the neuronal paths challenging in the regions near the eyes.

Several approaches have been reported to reduce this problem; however, none provide a complete solution. Techniques exist to reduce photon scattering (e.g., chemical clearing^19,27,34^ or using longer wavelengths in multi-photon fluorescence excitation^22,35^); however, unless these are used in combination with steps to reduce absorption (e.g., chemical bleaching), even the complete elimination of scattering will not reduce the attenuation caused by light-absorbing materials (such as pigmented blood or retinal cells). The “self-healing” properties of Bessel^36,37^ and Airy^38^ beams can create light sheets that are less susceptible to artifacts that are caused by localized regions of attenuating matter (absorbing or refracting); however, because this does not reduce artifacts due to attenuation of the emitted fluorescence, it reduces the problem rather than solving it. Alternatively, a variety of physical LSFM implementations attempt to access the sample from different angles to “see around” attenuating features. Multi-view^18,33^, multidirectional SPIM (mSPIM)^32,39^, and multi-arm^35,40–42^ LSFM systems can all reduce attenuation artifacts by illuminating and/or detecting light from different orientations and “bypass” the attenuating features of the sample, for example, adding a second light sheet from the opposite side. However, these are not universal solutions. A single attenuating region can be avoided by imaging around it, but more complex spatial distributions of absorbing materials can produce collections of shadows that cannot be removed in this way (e.g., see the schematic in Fig. 1a and the nerves in the interior of the embryonic mouse eye in Supplementary Fig. 1a).

Here, we propose a very different approach to solve this problem. Rather than trying to avoid attenuation, we aim to measure it. In an approach similar to that used by Vinegoni et al.^43^ to improve fluorescence OPT reconstructions, we explore whether an accurate 3D map of attenuation can be used to computationally correct the shadow artifacts generated by standard LSFM imaging. We and others have recently shown that OPT and multi-view LSFM are compatible imaging modalities that can be combined in a single hybrid system^44–47^—a combination we term OPTiSPIM (see Supplementary Fig. 4). This combination in a single instrument allows one to generate both high-resolution 3D fluorescence data (in SPIM mode) and 3D maps of the attenuating properties of the sample (in transmission OPT). OPTiSPIM has been used for a variety of samples, such as fixed adult murine organs (intestines, spinal cords^44^, and lymph nodes^46^), mouse embryos^45,46^, and live zebrafish embryos;^47^ however, thus far, it has been used solely to provide multiple independent channels of imaging. In contrast, in the current study, we explored a synergistic relationship by using one modality to improve the other. Specifically, we used the 3D map of attenuation created by OPT to computationally correct the artifacts in LSFM.

## Results

In LSFM, the camera directly images optical sections illuminated by the light sheet; thus, pixel values typically map directly into the 3D data set. In principle, the value recorded for each point in the tissue reflects the intensity of illumination and the concentration of fluorophores at that point. In reality, however, two sources of attenuation reduce this recorded value. First, the intensity of the light sheet itself may be reduced as it passes through the sample. Thus, different points in the tissue will receive different amounts of illumination. Second, fluorescently emitted light may also be absorbed on its route from the fluorophore to the camera. The two paths along which light may be absorbed are thus orthogonal to each other and cast shadows in two different directions, as shown in the images of an embryonic mouse head in Fig. 1. LSFM alone cannot visualize the structure causing the attenuation because the absorbing material does not fluoresce (Fig. 1c). However, a transmission OPT (tOPT) scan of the same sample reveals this unlabeled tissue to be the pigmented cells of the retina in developing eyes because the tomographic reconstruction calculates a spatial map of the attenuation coefficient (Fig. 1d, e), which we call *α*.

**Fig. 2:**
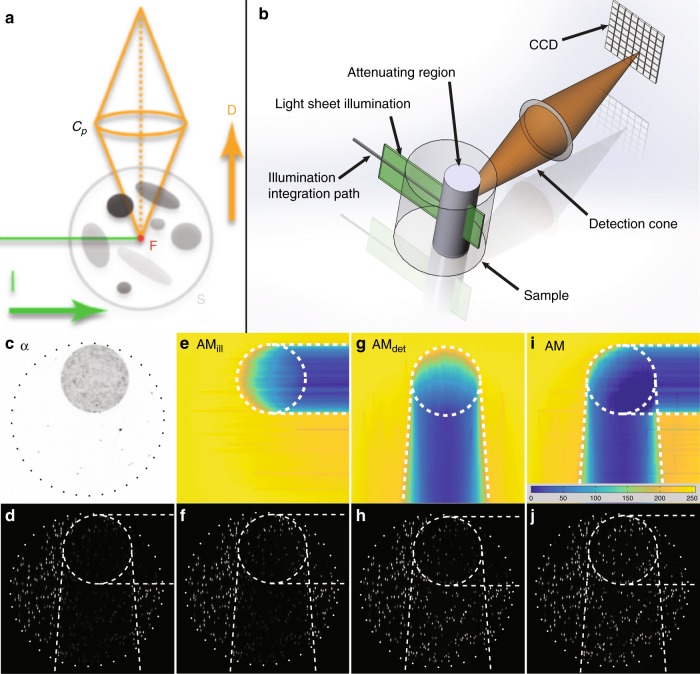
Principles of attenuation correction. **a** 2D schematic of the LSFM imaging process. The excitation light sheet (I, green) is incident on a fluorophore (F, red) within a sample containing attenuating components (S, gray). The detected fluorescence (D, orange) is collected by an objective lens (C_p_) and focused onto a camera (not shown). **b** The geometry of the ink and bead phantom: a cylinder with diluted ink (gray) is embedded in a larger cylinder of transparent agarose. Both contain fluorescent beads in the same concentration. The illuminating light sheet (green) is perpendicular to the detection cone (brown). **c**–**j** Virtual sections (perpendicular to the rotation axis) through the phantom: **c** attenuation coefficient, *α*. **d** Recorded fluorescent SPIM signal. **e** Attenuation map, AM, for illumination. **f** Fluorescent signal corrected for illumination attenuation. **g** AM for detection. **h** Fluorescent signal corrected for detection attenuation. **i** Combined AM for illumination and detection. **j** Fluorescent signal corrected for both illumination and detection attenuation. The dotted circle marks the transparent agarose cylinder, and the dashed circle marks the ink-containing agarose cylinder. Excitation illumination is from the left, and detection is towards the top of the images

A scheme to correct LSFM artifacts using OPT data must take a number of issues into account. At any given point in the sample, the reduction in light sheet intensity will depend on how much absorbing material is present between that point and the source of illumination. In a typical LSFM, the light sheet enters the sample on one side, and the effect of absorption on a given point is therefore an asymmetric function. For example, in Fig. 1c, the light sheet enters from the left, and therefore, the material to the right of a given voxel has no impact on the illumination of that voxel (voxels in the shadow appear darker because light-absorbing retinal pigments are to their left). One important consequence is that the illumination attenuation will be different for every point in the tissue, and a numerical correction must therefore be independently calculated for each voxel. This correction is based on the Beer–Lambert law and employs a path integral over attenuation coefficient values along a straight line from the illumination source to the imaged point (Fig. 2a, see the Materials and methods section, and Eq. 3).

Calculating the correction for the emitted fluorescent light is similar, but there is one extra complication. For LSFM detection, the emitted light is collected over the entire aperture cone of the detection objective lens (*C*_p_ in Fig. 2a). Similar to illumination, we assume that the emitted light travels along straight paths (neglecting scattering and refraction); however, unlike illumination, multiple paths are possible across the 3D volume of the aperture cone (see Fig. 2b). In a sample of non-uniform attenuation, each distinct path within the detection cone may pass through regions of different attenuation. The effect of the attenuation on the fluorescence emitted by a fluorophore at {*x*,*y*,*z*} is therefore the integral over all the paths within the detection cone (see the Materials and methods section and Eq. 6).

As Eq. 6 requires the solution of a triple integral, whereas illumination AM (AM_ill_) involves only a single integral (Eq. 3), the determination of detection AM (AM_det_) is significantly more computationally intensive. While the attenuation coefficient map, *α*, must only be calculated once for a given sample, the attenuation maps (AMs) must be recalculated for each orientation of scanning if multi-view LFSM is performed (because of the asymmetric nature of the illumination and detection paths). See Supplementary Fig. 5.

Figure 2c–j illustrates a test of the proposed correction method using a phantom consisting of fluorescent beads and ink suspended in agarose with a well-defined geometry. The experimental configuration is sketched in Fig. 2b; the inputs for the correction are in Fig. 2c (the absorption coefficient, *α*, reconstructed from the tOPT scan) and Fig. 2d (the raw SPIM fluorescence data). Individual correction of both the illumination (using Eq. 5, Fig. 2e, f) and detection (using Eq. 6 and Fig. 2g, h) attenuation artifacts are shown, as well as the complete correction (Fig. 2i, j).

**Fig. 3:**
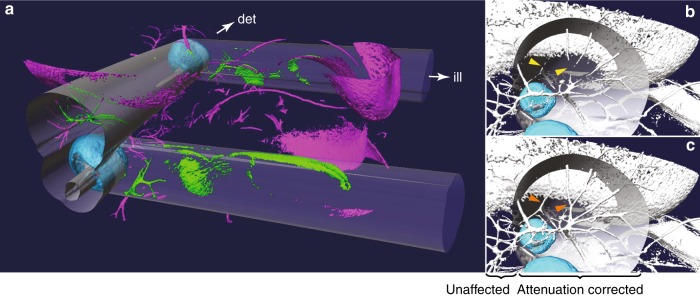
The use of OPT attenuation maps to correct artifacts in fluorescent SPIM data—isosurfaces. The directions of illumination (ill) and detection (det) are indicated; the sample is the E12.5 embryonic mouse head from Fig. 1. **a** Overview of the region of the sample that experiences significant attenuation from eye pigmentation (cyan). The volumes strongly affected by attenuation are indicated by the gray quasi-cylindrical translucent structures (illumination attenuation extends roughly to the right from the eyes; detection attenuation is approximately into the plane of the image). Magenta: Unattenuated fluorescence; green: regions of fluorescence that experienced significant attenuation and were corrected. **b**, **c** Visualizations of the sample looking into the detection direction behind one eye (cyan): **b** before attenuation correction and **c** after correction. Yellow arrowheads indicate missing structures in the uncorrected data that are restored after correction (orange arrowheads)

We chose to explore whether this new approach could correct the SPIM artifacts seen in Fig. 1. Fig. 1b shows a surface rendering of the sample, which gives an idea of the relative positions of the eyes and neuronal structures. Because it provides an overview of the entire head, the attenuation artifacts caused by the eye pigmentation are not readily apparent. In Fig. 3a, we have therefore rendered a sub-volume of the fluorescent structures of the head in Fig. 1b, where the shadow artifacts are now visible, both to the right of the eye (where the fluorophores are only weakly excited because the eye pigments block the illuminating light sheet) and behind the eye with respect to the detection direction (where the emitted fluorescence is blocked from reaching the detection optics by the pigmentation). Fig. 1d, e show the reconstruction of the eye pigmentation from a tOPT scan in the region indicated by the red box in Fig. 1c and the overlay of the pigmentation and the fluorescence signal imaged in SPIM mode, respectively. The shadow artifacts in the fluorescence data are well aligned with the eye pigmentation.

Thus, we thus explored if it would be possible to use our knowledge of the 3D pigment distribution to correct the fluorescence shadow artifacts. We implemented a numerical 3D solution for Eqs. 4 and 6 that took as inputs the distribution of the attenuator (such as the eye pigmentation in Fig. 1) and the geometry of the SPIM’s illumination and detection processes (see the Materials and methods section for details). The resulting “AM” could then be numerically inverted and multiplied by the measured fluorescence signal to correct/amplify the fluorescence signal in regions that had suffered attenuation from the pigmentation.

The result of applying this attenuation correction to the data in Fig. 1 is presented in Fig. 3. Fig. 3a depicts the volume of the embryonic mouse head in which significant attenuation occurs. The two types of attenuation shadows (illumination to the right of the eyes and detection perpendicular to the illumination) are rendered as transparent (quasi) cylinders emanating from the absorbing structures, the pigmented retinas (cyan). The raw SPIM intensities (magenta) reveal a considerable reduction in signal within the cylinders; however, our method provided recovery of this signal (green). The attenuation artifacts and their correction can be more clearly observed by taking the viewpoint looking towards the camera, as depicted both without (Fig. 3b) and with (Fig. 3c) correction of the attenuation effects. Without correction, the nerve structures are fragmented and incomplete due to the weakened signal reaching the camera from behind the eye. In contrast, after correction was applied, structures that were too dim to be visible are clearly visible, and the correct intact nerve arrangement can be segmented. Importantly, intensity changes are only observed in the region behind the eye where its shadow is cast; in the unaffected region (outside the cylindrical shadow) where neither illumination nor detection experience significant attenuation, there is virtually no change in the observed fluorescence.

**Fig. 4:**
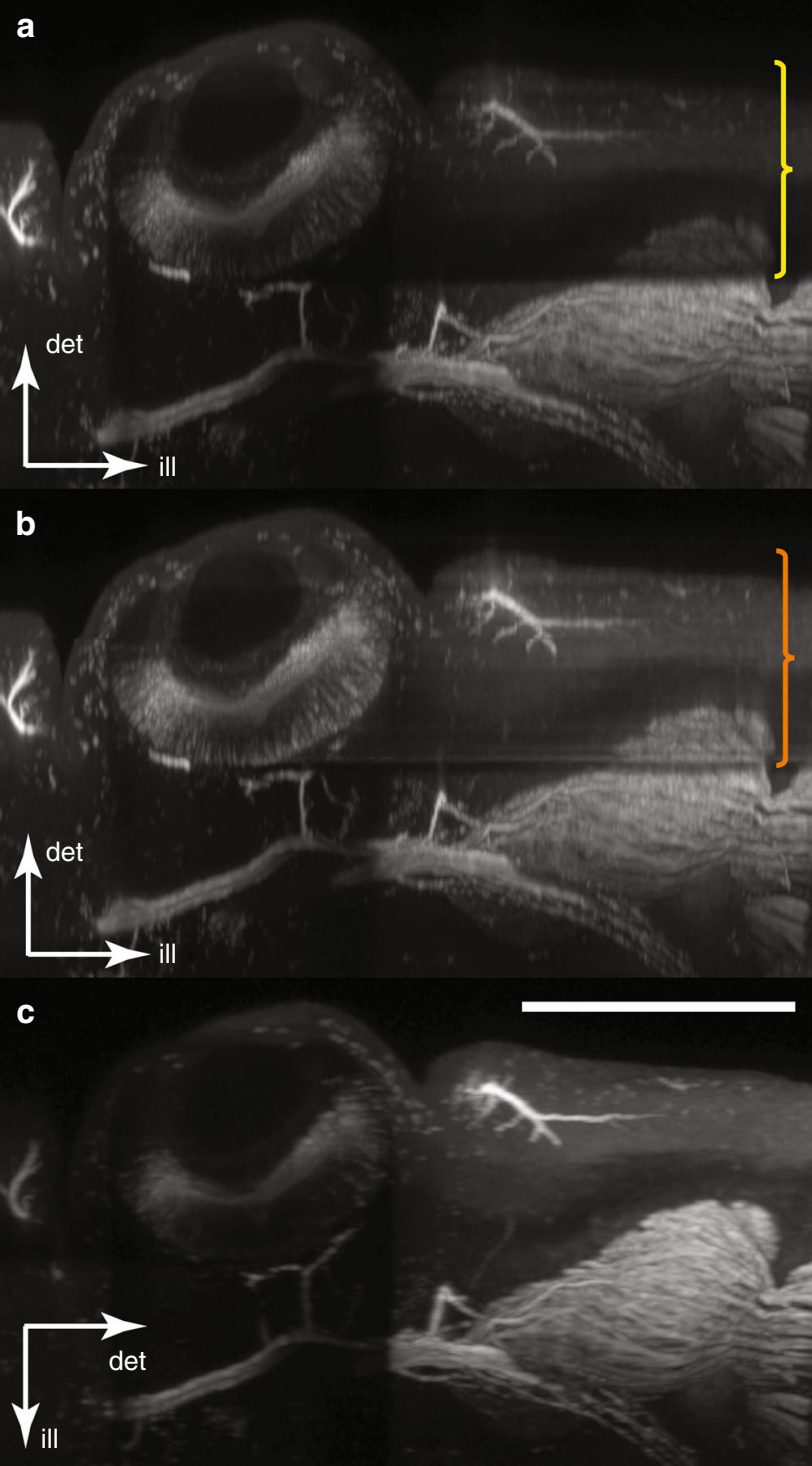
The use of OPT attenuation maps to correct artifacts in fluorescent SPIM data—optical sections. The directions of illumination (ill) and detection (det) are indicated; the sample is the E12.5 embryonic mouse head from Figs. 1 and 3. Optical sections from the fluorescent SPIM data set of approximately the same region shown in Fig. 1e. Images are shown before (**a**) and after (**b**) attenuation correction. The yellow bracket indicates the region for which the excitation is strongly affected by attenuation in (**a**) but is well corrected in (**b**) (orange bracket). **c** The same region imaged after rotation of the sample by 90°. The fluorescence to the right of the eye is unattenuated and serves as a control to illustrate what the corrected data in **b** should look like. Scale bar: 500 µm

To assess the degree of attenuation more directly, we examined the same virtual section shown in Fig. 1e and compared it without (Fig. 4a) and with (Fig. 4b) correction. The region below the eye where detection was attenuated and, in particular, to the right of the eye where illumination was reduced are considerably brighter after correction. To determine whether these corrected fluorescence levels are an accurate representation of the actual fluorophore distribution, we rotated the sample by 90° and re-scanned it; the result is shown in Fig. 4c, where the new illumination and detection directions are indicated. In this orientation, neither the illumination nor the detection in the region to the right of the eye experience significant attenuation and can therefore be used as the standard with which to compare our corrected attenuation (Fig. 4b). The greyscale levels in the shadowed region (orange bracket in Fig. 4b) are boosted back up to the correct levels. A residual thin, dark “stripe” remains in the corrected data, extending from the lower edge of the eye along the illumination axis (to the right of the eye in Fig. 4b). This is likely due to the very strong absorption experienced by the illumination light passing through this region (as shown in Fig. 1e, the illumination passing through the lower edge of the eye will traverse the largest region of pigmented tissue). As discussed in the Materials and methods section, the accuracy of the determination of the absorption coefficient from tOPT data becomes challenging for regions of extreme absorption. An alternative cause of the stripe artifact observed in Fig. 4b may be residual refractive index variations in the sample that were not completely eliminated by the chemical clearing process.

For a second example of attenuation correction, see Supplementary Text Section 1 and Supplementary Fig. 3, which describe and show a 3D reconstruction of a mouse lymph node that was stained using the standard non-fluorescent in situ hybridization protocol to reveal the gene expression patterns.

## Discussion

As has been observed repeatedly in the past^22,32–34,36^, LSFM can suffer from optical artifacts caused by non-fluorescent absorbing materials. There is no intrinsic solution for this issue in LSFM because such materials cannot be directly characterized by fluorescence imaging. We demonstrated that our novel method of attenuation correction in LSFM using OPTiSPIM data can significantly reduce these artifacts, bringing back biological details to the image (such as fine nerve structures) and recreating overall greyscale levels very similar to the unattenuated control. It achieves this improvement in a positive manner by measuring the absorption, rather than by trying to avoid it. As shown by the comparison of the embryonic mouse head in Fig. 4a–c, our corrected version of the data (Fig. 4b) is clearly much more similar to the unattenuated region of the control image (Fig. 4c) than to the raw data (Fig. 4a). Similarly, for the in situ stained lymph nodes in Supplementary Fig. 3, the data after correction gives a more accurate representation of the lymph node than does the uncorrected data.

Some residual shadow artifacts may remain even after correction is applied (the dark horizontal line extending to the right from the bottom of the eye in Fig. 4b and the vertical streaks in Supplementary Fig. 3d). These generally occur when the attenuation reduces the fluorescence signal down to or below background levels. Because our correction enhances a “real” signal, our implementation (see the Materials and methods section and Eq. 11) explicitly suppresses amplification in these regions. A direct application of the Beer–Lambert law (see the Materials and methods section and Eq. 7) would result in amplified background/noise in these regions. Our use of Eq. 11 rather than Eq. 7 was motivated by the “first, do no harm” principle: in regions where we suspect the measured signal to be simply background or noise, rather than amplify this “signal,” we chose to suppress the amplification that the Beer–Lambert law would suggest and retain the raw, measured values. See Supplementary Text Section 2 and Supplementary Fig. 2 for further information. However, clearly, such artifacts remain in only a small region of the corrected image, and there may be approaches for reducing or removing this issue in the future.

One of the advantages of the attenuation correction system presented here is that it can be used as a compliment to previous methods that have been developed to avoid attenuation artifacts. Both mSPIM and multi-view LSFM are capable of reducing these artifacts, but their effectiveness in samples with complex geometries (see the schematic in Fig. 1a) can be compromised. In this paper, we show that our method is compatible with single-sided mSPIM (Eq. 4 in the Materials and methods section describes this), and the generalization to multi-sided illumination and multi-view imaging should be straightforward. Although “self-healing” light sheets (e.g., using Bessel or Airy beams) can help to reduce *illumination* artifacts, neither they nor the mSPIM technique can deal with artifacts caused by attenuation of the *detected* fluorescence. Since the OPTiSPIM-based method described here depends on *measurement and correction of the attenuation* as opposed to approaches that attempt to “view around” attenuating features, we expect that it will serve as a complimentary addition to self-healing light sheets and mSPIM. Table 1 summarizes important approaches that have been described to combat attenuation artifacts in LSFM data with some of their benefits and limitations.

**Table 1:** Methods to alleviate attenuation in LSFM

Method	Benefits	Limitations	References
Chemical clearing	• Reduces optical scattering • Compatible with other (non-LSFM) optical microscopy methods	• Not compatible with live imaging • Complete clearing difficult with large/dense samples • Protocols can be slow (weeks) and use toxic reagents • Most protocols do not reduce absorption	^19, 27, 34^
Purely computation methods	• Require no extra imaging hardware/data acquisition	• Discrepancies between theory used in corrections and practical imaging conditions can introduce artifacts	Supp. Mat. 3, Supp. Mat. 4
Multi-photon excitation	• Reduced scattering of excitation light • Reduced photo-toxicity • “All-optical” method	• Does not correct artifacts in the detected fluorescence • Depth of imaging limited (<1 mm)	^22, 35^
“Self-healing” excitation	• “All-optical” method	• Does not correct artifacts in the detected fluorescence	^35, 36, 37^
Multi-view SPIM	• Can also improve resolution	• Sequential view acquisition (slow) • Computational post-processing required	^18, 33^
mSPIM	• “All-optical” method • Straightforward, economical implementation	• Does not correct artifacts in the detected fluorescence	^32, 39^
Multi-arm LSFM	• High data acquisition rates	• Requires complicated, expensive hardware	^40– 42, 43^
OPTiSPIM	• Correct regions where absorption cannot be avoided • 3D attenuation maps available “for free”	• Computational post-processing required • (currently) custom-built setup required • OPT of small samples (<100 µm) may require methods to extend the depth of field	Present work

The samples considered in this study were either optically cleared (biological specimens) or were intrinsically very low scattering (the fluorescent beads in aqueous agarose, Fig. 2). In these cases, as discussed in the Materials and methods section, Eq. 2 provides a good model of the attenuation. However, many applications of LSFM involve imaging of living samples that cannot be optically cleared by the methods we employed. These live samples can introduce two types of problems that were avoided in this study. First, our method requires generating both LSFM and OPT scans of the sample, which will limit the temporal resolution that is achievable (compared to LSFM alone). Although this may be a significant issue when high speed is critical, tOPT has an advantage because it does not rely of fluorescence contrast and thus very short exposure times can be used. Bassi et al.^47^ demonstrated combining LSFM and tOPT for living zebrafish embryos; thus, at least in this widely used model organism, we expect that our method will be applicable. The second issue that may arise with our method when imaging live, uncleared biological samples is that refraction/scattering may be significant so that Eq. 2 is not valid. In this case, our attenuation model (and tOPT apparatus) would have to be modified to account for (and quantify) the sample’s refractive index variations. Such a method might be possible by implementing a diffraction tomography system;^48^ however, this is beyond the scope of this study.

In principle, our method for correcting attenuation artifacts can be applied to other microscopy techniques besides LSFM, such as confocal microscopy. Computationally, all that would be required would be a change in the integration paths in Eqs. 5 and 6 (see the Materials and methods section). However, we are unaware of any imaging system besides the OPTiSPIM that allows the collection of both fluorescence data and a map of the attenuating features of the sample.

In summary, we present a novel method for the correction of attenuation artifacts in LSFM that takes advantage of two different imaging modalities: (1) the measurement of fluorescence data (via the SPIM mode of OPTiSPIM) and the distribution of the attenuation coefficient (via tOPT) and (2) the computational correction of the former by using a physical model based on the latter. Our method is easy to incorporate into most LSFM platforms that allow sample rotation. Importantly, the proposed method is compatible with previously published techniques for attenuation artifact correction and can act as a complement to techniques such as mSPIM and multi-view LSFM imaging.

## Materials and methods

Imaging was performed using the OPTiSPIM setup described in Mayer et al^46^. Briefly, for SPIM illumination, a single arm employing a cylindrical lens to create the light sheet was used. Detection was via a CCD camera coupled to a telecentric optical lens system. The sample was mounted from above and suspended in an imaging chamber located at the intersection of the illumination and detection arms. Within the imaging chamber, the sample can be translated along the three orthogonal spatial axes and rotated about the vertical axis; these degrees of freedom permit both OPT (rotational) and SPIM (translational) scanning. A schematic of the setup is shown in Supplementary Fig. 4.

### Fundamentals of attenuation correction

We used tOPT to reconstruct the 3D map of the attenuation coefficient of the sample. OPT was designed so that a raw image measured in transmission mode is the shadow projection of the sample onto the camera. Because the diffraction limits both the imaging resolution and the depth of field, without using techniques to extend the depth of field, OPT is generally best suited to sample sizes that are more than ~100 µm (see Supplementary Text Section 4 for a discussion of this issue)^5^. Quantitative reconstruction of the attenuation requires that some light be transmitted through the sample. For regions that are completely opaque, no information is available.

To correct for attenuation, we first consider the Beer–Lambert law:^49^

1 $$I = I_0 \cdot \exp \left( { - \alpha \cdot x} \right)$$

where *I*_0_ is the incident intensity, *α* is the attenuation coefficient, and *x* is the thickness of the object. This formula represents the case for spatially uniform attenuation; in a more general case where the attenuation can vary spatially, the product *α⋅x* becomes a path integral along a light ray:

2 $$I\left( {\mathop{r}\limits^{\rightharpoonup} } \right) = I_{0} \cdot \exp \left( {\mathop {\int}\limits_{ - \infty }^{\mathop{r}\limits^{\rightharpoonup} } { - \alpha \left( {\mathop{s}\limits^{\rightharpoonup} } \right) \cdot {\mathrm{d}}\mathop{s}\limits^{\rightharpoonup} } } \right)$$

where $\alpha \left( {\mathop{r}\limits^{\rightharpoonup} } \right)$ is the attenuation coefficient at position $\mathop{r}\limits^{\rightharpoonup}$ in the sample. Here, we assume that the imaging processes can be described by a ray optics model, that is, diffraction and refraction are not taken into account. This may be responsible for minor artifacts in the corrected data when imaging at a high resolution using high NA optics or in samples with significant variations of refractive indices (e.g., see the “stripe” artifact extending from the bottom of the eye in Fig. 4b and the discussion in the Results section).

Equation 2 (and the following equations that are based on it) contains the implicit assumption that photons attenuated by the sample do not contribute to the image formation process. This will be the case when the attenuation is due to *absorption* during tOPT imaging of the sample (we neglect the possibility of significant fluorescence emission subsequent to the absorption, which can be eliminated by spectral filtering). Attenuation via *scattering* can also be modeled by Eq. 2, provided that the scattered light is not collected by the imaging optics. However, samples that can scatter light in such a way that it does contribute to the imaging process (e.g., back-reflected light or diffuse scattering in turbid media) will not be correctly modeled by Eq. 2. A correct treatment of these types of samples would require a more detailed model of the scattering process, which is beyond the scope of this paper. However, even with this restriction, there is a wide range of biological samples for which attenuation can be corrected via this method.

We take advantage of the fact that a reconstructed tOPT data set is a good approximation to the attenuation coefficient, $\alpha \left( {\mathop{r}\limits^{\rightharpoonup} } \right)$, of the sample^50^. Thus, the calculation of the effect of the attenuation on the fluorescence SPIM image—what we term the AM—can be based on the tOPT reconstruction, $\alpha \left( {\mathop{r}\limits^{\rightharpoonup} } \right)$. This approximation may fail for very strongly absorbing regions of the sample: as the measured value of $I\left( {\mathop{r}\limits^{\rightharpoonup} } \right)$ in Eq. 2 approaches zero, the back-projection algorithm that is used to calculate $\alpha \left( {\mathop{r}\limits^{\rightharpoonup} } \right)$ becomes less accurate.

The formation of a fluorescence image can be thought of as the combination of two processes: light from the excitation source (in the case of LSFM, the light sheet) must propagate to (and excite) the fluorophore to be imaged, and the light emitted by the fluorophore must propagate to the detector (for LSFM, a camera). This geometry is sketched in Fig. 2b.

We first consider the simpler process of LSFM illumination: the light sheet is modeled as a non-diffracting plane of light, which we consider to be propagating along the *x*-axis of the microscope. In this case, we rewrite Eq. 2 to define the illumination AM,${\mathrm{AM}}_{\mathrm{ill}},$:

3 $${\mathrm{AM}}_{\mathrm{ill}}\left( {x,y,z} \right) = \frac{{I\left( {x,y,z} \right)}}{{I_0}} = \exp \left( { - \mathop {\int}\limits_{ - \infty }^x {\alpha \left( {x{\prime},y,z} \right) \cdot {\mathrm{d}}x{\prime}} } \right)$$

Integration is performed up to point *x* where the fluorophore under consideration is located. We approximate the light sheet as an infinitesimally thin plane of light:

4 $$I\left( {x,y,z} \right) = I_0 \cdot \delta \left( z \right) \cdot H\left( {y,\Delta y} \right)$$

where $\delta \left( z \right)$ is a delta-function, $$\begin{array}{l}H\left( {y,\Delta y} \right) = 1,\quad\,\left| y \right| < \Delta y\\ \quad \quad \quad \,\,\, = 0,\quad{\mathrm{otherwise}}\end{array}$$, and, $2\Delta y$ is the height of the light sheet.

If a resonant scan mirror (RSM) is used to tilt the light sheet, as in mSPIM^32^, we can modify Eq. 3 to account for this:

5 $$\begin{array}{l}{\mathrm{AM}}_{\mathrm{ill}}\left( {x{\mathrm{,}}y{\mathrm{,}}z} \right) = \frac{1}{{\varphi _{\mathrm{max}} - \varphi _{\mathrm{min}}}}\\ \quad {\int}_{\varphi _{\mathrm{min}}}^{\varphi _{\mathrm{max}}} {\exp \left( -{\mathop {\int}\limits_{ - \infty }^x {{\mathrm{Rot}}\left( {\alpha \left( {x\prime ,y,z} \right),\varphi \prime } \right)} \cdot {\mathrm{d}}x\prime } \right) \cdot {\mathrm{d}}\varphi \prime } \end{array}$$

where {*φ*_min_, *φ*_max_} is the range of angles through which the light sheet is scanned by the RSM, and ${\mathrm{Rot}}\left( {\alpha ,\varphi } \right)$ denotes a function that rotates the 3D distribution of the attenuation coefficient, $\alpha \left( {\mathop{r}\limits^{\rightharpoonup} } \right)$, by angle *φ* in the plane of the light sheet (the *xz* plane). For a static light sheet with *φ*_min_ = *φ*_max_ = 0, Eq. 5 reduces to Eq. 3.

For LSFM detection, the emitted light is collected over the entire aperture cone of the objective lens used for detection (see Fig. 2b). As with the illumination, we assume that the emitted light travels along straight paths (neglecting scattering and refraction), but in a sample of non-uniform attenuation, each distinct path within the detection cone may pass through regions with different attenuation. The effect of the attenuation on the fluorescence emitted by a fluorophore at {*x*,*y*,*z*} is therefore the integral over all the paths within the detection cone. For convenience, we perform the integral using polar coordinates centered at {*x*,*y*,*z*}:

6 $${\mathrm{AM}}_{\mathrm{det}}\left( {x,y,z} \right) = \frac{1}{{2\pi \vartheta _{\mathrm{max}}}}{\int}_0^{\vartheta _{\mathrm{max}}} {{\int}_{ - \pi }^\pi {\exp \left( { - {\int}_0^{r_{\mathrm{max}}} {\alpha \left( {r,\varphi ,\vartheta } \right) \cdot {\mathrm{d}}r} } \right) \cdot {\mathrm{d}}\varphi } \cdot {\mathrm{d}}\vartheta }$$

As Eq. 6 requires a triple integral, whereas ${\mathrm{AM}}_{\mathrm{ill}}$ requires only a single (Eq. 3) or a double (Eq. 5) integral, the determination of ${\mathrm{AM}}_{\mathrm{det}}$ is the more computationally intensive calculation.

Because excitation and emission are independent processes, once ${\mathrm{AM}}_{\mathrm{ill}}$ and ${\mathrm{AM}}_{\mathrm{det}}$ have been calculated, the total AM of the complete LSFM imaging process is given by their product:

7 $${\mathrm{AM}} = {\mathrm{AM}}_{\mathrm{ill}} \cdot {\mathrm{AM}}_{\mathrm{det}}$$

Having determined the AM, we next consider the form of the detected fluorescence signal that will be generated by an LSFM measurement. To a good approximation, this is given by

8 $$F_{\mathrm{det}} = {\mathrm{AM}} \cdot F_0 + B$$

where *F*_0_ is the “real” signal (that we want to recover), and *B* is the background signal. *B* represents the fact that during the fluorescence imaging process the measured signal may have received a contribution that is not directly related to the concentration of the fluorophore at point {*x*,*y*,*z*} being imaged. Examples of processes that would contribute to this contamination are background room lights that are not completely blocked or thermal noise in the CCD detector. In practice, *B* can be determined by measuring the mean detected signal in a region of a fluorescence image outside the sample (where it is known that there are no fluorophores present). This equation for $F_{\mathrm{det}}$ is easily inverted to solve for *F*_0_:

9 $$F_0 = (F_{\mathrm{det}} - B)/{\mathrm{AM}}$$

Although the variables in the above equations (and those that follow) are often 3D matrices, the functions $X \cdot Y$ (multiplication), $X/Y$ (division), and $X^{ - 1}$ (inverse) are performed element-wise rather than as matrix operations. Supplementary Fig. 5 illustrates the processing steps involved in collecting and processing the data graphically. Fig. 2c–j depicts these steps using experimentally measured data from a simple fluorescent beads-and-ink phantom.

Although eq. 9 is theoretically valid, we found in practice that there are several modifications to it that result in more stable and accurate corrections of attenuation artifacts in LSFM images.

### Attenuation correction in the presence of background/noise

First, considering the form of Eqs. 3–7, clearly, for a finite *α*, the value(s) of the AM(s) will fall within the range of $0\, < \, {\mathrm{AM}} \le 1$. ${\mathrm{AM}}(x,y,z) = 1$ implies that the fluorescent signal from point {*x*,*y*,*z*} in the sample is unaffected by attenuation, and the smaller the value of AMI, the more the fluorescence has been attenuated.

For Eq. 9 to be physically meaningful, we require that the background *B* be positive and that $F_{\mathrm{det}} \ge B$. Ideally, these conditions will be satisfied; however, in real measurements, noise may play a significant role. To investigate this role further, we re-formulate Eq. 9 to explicitly account for errors/uncertainties in the various parameters:

10 $$\begin{array}{*{20}{c}} {F_0 \pm \Delta F_0 = \frac{{\left( {F_{\mathrm{det}} \pm \Delta F_{\mathrm{det}}} \right) - \left( {B \pm \Delta B} \right)}}{{{\mathrm{AM}} \pm \Delta {\mathrm{AM}}}}} \\ {F_0 \pm \Delta F_0 = \frac{{\left( {F_{\mathrm{det}} - B} \right) \pm \sqrt {\Delta F_{\mathrm{det}}^2 + \Delta B^2} }}{{{\mathrm{AM}} \pm \Delta {\mathrm{AM}}}}} \end{array}$$

Because our measurements of $F_{\mathrm{det}}$, *B*, and ${\mathrm{AM}}$ are independent, the relative error in the calculation of the signal *F*_0_ is

11 $$\frac{{\Delta F_0}}{{F_0}} = \sqrt {\frac{{\Delta F_{\mathrm{det}}^2 + \Delta B^2}}{{\left( {F_{\mathrm{det}} - B} \right)^2}} + \frac{{\Delta {\mathrm{AM}}^2}}{{{\mathrm{AM}}^2}}}$$

This equation indicates that the error in our calculation of the real signal *F*_0_ will be large when $F_{\mathrm{det}} - B$ or ${\mathrm{AM}}$ is small, that is, when either the detected signal is close to the background level $\left( {F_{\mathrm{det}}\sim B} \right)$ or when the attenuation is large $\left( {{\mathrm{AM}} \to 0} \right)$. Therefore, we chose to modify Eq. 9 to avoid the high-error regime as follows. We first rewrite Eq. 9 as

12 $$\begin{array}{l}F_0 = \left( {F_{\mathrm{det}} - B} \right) - \left( {F_{\mathrm{det}} - B} \right) + \left( {F_{\mathrm{det}} - B} \right) \cdot \frac{1}{{\mathrm{AM}}}\\ F_0 = \left( {F_{\mathrm{det}} - B} \right) + \left( {F_{\mathrm{det}} - B} \right) \cdot \left( {\frac{1}{{\mathrm{AM}}} - 1} \right)\end{array}$$

Written this way, the “real” signal *F*_0_ is composed of the raw data $\left( {F_{\mathrm{det}} - B} \right)$ plus a term that takes attenuation into account. We next introduce a weighting factor, *S*, to the second term (the one that compensates attenuation):

13 $$F_{\mathrm{est}} = \left( {F_{\mathrm{det}} - B} \right) + S \cdot \left( {F_{\mathrm{det}} - B} \right) \cdot \left( {\frac{1}{{\mathrm{AM}}} - 1} \right)$$

where $F_{\mathrm{est}}$ is now our estimate of the real signal, *F*_0_. Thus, we define *S* so that when our attenuation correction is trustworthy, *S* ≈ 1, and Eq. 13 is a good approximation to Eq. 9. However, in situations in which Eq. 9 may just amplify the noise, we want to have *S* ≈ 0 so that $F_{\mathrm{est}} \approx F_{\mathrm{det}} - B$.

The weighting factor, *S*, that we use in Eq. 13 should be our best estimate of the likelihood that our measured signal, $F_{\mathrm{det}}$, is primarily real and not background, that is, we chose *S* to be

14 $$S = \frac{{{"} {\mathrm{detected}}\,{\mathrm{signal}}\,{\mathrm{in}}\,{\mathrm{the}}\,{\mathrm{absence}}\,{\mathrm{of}}\,{\mathrm{background}}{"} }}{{{"} {\mathrm{detected}}\,{\mathrm{signal}}{"} }}$$

to satisfy the above requirements. From Eq. 9, this becomes

15 $$\begin{array}{l}S = \frac{{\mathrm{AM} \cdot F_0}}{{F_{\mathrm{det}}}}\\ {\hskip3.3pc}S = \frac{{{\mathrm{AM}} \cdot \left( {(F_{\mathrm{det}} - B)/{\mathrm{AM}}} \right)}}{{F_{\mathrm{det}}}}\\ S = \frac{{F_{\mathrm{det}} - B}}{{F_{\mathrm{det}}}}\end{array}$$

Substituting Eq. 15 into Eq. 13 and simplifying:

16 $$\begin{array}{l}F_{\mathrm{est}} = (F_{\mathrm{det}} - B) + \frac{{F_{\mathrm{det}} - B}}{{F_{\mathrm{det}}}} \cdot \left( {\frac{1}{{\mathrm{AM}}} - 1} \right) \cdot (F_{\mathrm{det}} - B)\\ F_{\mathrm{est}} = (F_{\mathrm{det}} - B) + \frac{{\left( {F_{\mathrm{det}} - B} \right)^2}}{{F_{\mathrm{det}}}} \cdot \left( {\frac{{1 - {\mathrm{AM}}}}{{\mathrm{AM}}}} \right)\\ F_{\mathrm{est}} = \left( {F_{\mathrm{det}} - B} \right) \cdot \left[ {1 + \frac{{\left( {F_{\mathrm{det}} - B} \right) \cdot \left( {1 - {\mathrm{AM}}} \right)}}{{\mathrm{AM} \cdot F_{\mathrm{det}}}}} \right]\end{array}$$

This is the equation that we have implemented to perform our attenuation correction calculations. For $F_{\mathrm{det}} > > B$ (i.e., when the measured signal is substantially greater than the background and we can trust our method of attenuation correction), Eq. 16 reduces to Eq. 9. Additionally, in the low-attenuation regime where ${\mathrm{AM}} \to 1$, Eq. 16 becomes $F_{\mathrm{est}} = F_{\mathrm{det}} - B$ as expected.

To estimate the value of *B* for a given experiment, we take “dark” images of the sample, with all filter, camera, and other settings identical to those for imaging, but with the light sheet power set to zero. In principle, the average signal level in this “dark” image can be taken as the value of *B*. In practice, we found better results were obtained (i.e., better suppression of noise amplification) by setting *B* equal to the mean of the “dark” image signal + 1 standard deviation of the signal because this gives a more conservative estimate of the background level.

### The spectral dependence of attenuation

Another issue that has not been explicitly accounted for in either Eq. 9 or Eq. 16 is that, generally, we cannot expect $\alpha \left( {\mathop{r}\limits^{\rightharpoonup} } \right)$ and thus AM to be wavelength-independent. The extent to which this will have a significant effect on our results will depend on the properties of the attenuating material. The ink used in the bead phantom (Fig. 2c–j) does not have a strong spectral dependence, at least in the visible region of the spectrum; however, the NBT/BCIP staining used in the lymph nodes (Supplementary Fig. 3) does have a noticeable chromaticity. This means that when we perform tOPT to generate the AM, we should ensure that the wavelengths used are appropriate. For example, because of the Stokes shift between the excitation and emission wavelengths in fluorescence, ideally, ${\mathrm{AM}}_{\mathrm{ill}}$ and ${\mathrm{AM}}_{\mathrm{det}}$ will each be generated from their own $\alpha _{\mathrm{ill}}$ and $\alpha _{\mathrm{det}}$ at the appropriate wavelengths. In practice, we achieved this by using a halogen lamp as a transmission source and by putting the appropriate filters in the detection path (see the Scanning section below). This results in a slight modification to Eqs. 5 and 6 into forms

17 $$\begin{array}{l}{\mathrm{AM}}_{\mathrm{ill}}\left( {x,y,z} \right) = \frac{1}{{\varphi _{\mathrm{max}} - \varphi _{\mathrm{min}}}}\\ \quad {\int}_{\varphi _{\mathrm{min}}}^{\varphi _{\mathrm{max}}} {\exp \left( { - \mathop {\int}\limits_{ - \infty }^x {\mathrm{Rot}\left( {\alpha _{\mathrm{ill}}\left( {x\prime ,y,z} \right),\varphi \prime } \right)} \cdot {\mathrm{d}}x\prime } \right) \cdot {\mathrm{d}}\varphi \prime } \end{array}$$

and

18 $$\begin{array}{l}AM_{\mathrm{det}}\left( {x,y,z} \right) = \\ \quad \frac{1}{{2\pi \vartheta _{\mathrm{max}}}}{\int}_0^{\vartheta _{\mathrm{max}}} {{\int}_{ - \pi }^\pi {\exp \left( { - {\int}_0^{r_{\mathrm{max}}} {\alpha _{\mathrm{det}}\left( {r,\varphi ,\vartheta } \right) \cdot {\mathrm{d}}r} } \right) \cdot {\mathrm{d}}\varphi } \cdot {\mathrm{d}}\vartheta } \end{array}$$

where the wavelength dependence of the attenuation coefficients is explicit.

Because of hardware constraints, it was not possible to scan the embryonic mouse head (Figs. 1, 3 and 4) using the halogen lamp, and thus we measured our $\alpha \left( {\mathop{r}\limits^{\rightharpoonup} } \right)$ at a wavelength (660 nm) that was significantly different from either the excitation (488 nm) or emission (~525 nm) wavelengths of the fluorophores in the sample. We realized that in many OPTiSPIM setups, it may not be possible to generate spectrally accurate $\alpha \left( {\mathop{r}\limits^{\rightharpoonup} } \right)$. Thus, we decided to adapt our procedure to take this into account. To make the problem tractable we assumed that, in spectral terms, there is only one important type of attenuating substance in the sample. This is a reasonable assumption in the case of the lymph node shown in Supplementary Fig. 3, where the attenuation is predominantly caused by NBT/BCIP staining, or in the case of the embryonic mouse head in Figs. 1, 3 and 4, where the only significant attenuation is from the eye pigmentation. It would probably not be valid, for example, if we performed in situ NBT/BCIP staining on the mouse head, which would then contain two strong sources of attenuation with presumably uncorrelated spectral properties.

For samples with a single attenuating species, we assumed that a shift in wavelength will result in a rescaling of the attenuation coefficient, but that this rescaling is independent of the position in the sample. Thus, instead of directly applying Eqs. 17 and 18, we first applied the transformations

19 $$\alpha _{\mathrm{ill}} = K_{\mathrm{ill}} \cdot \alpha _{\mathrm{measured}}$$

and

20 $$\alpha _{\mathrm{det}} = K_{\mathrm{det}} \cdot \alpha _{\mathrm{measured}}$$

where $K_{\mathrm{ill}}$ and $K_{\mathrm{det}}$ are factors of proportionality between the attenuation at the measured wavelength and at the illumination and detection wavelengths, respectively.

See also Supplementary Text Section 3.

## Electronic supplementary material

Supplementary Material
