# Supplementary material for: Attenuation artifacts in light sheet fluorescence microscopy corrected by OPTiSPIM
Source: Light Sci Appl. 2018 Oct 3;7:70. doi: 10.1038/s41377-018-0068-z (PMC6168557; doi:10.1038/s41377-018-0068-z)
Supplement: Supplementary file 1 — Supplementary Material [file 41377_2018_68_MOESM1_ESM.docx]

Supplementary Material for

full title: ***Attenuation Artifacts in Light Sheet Fluorescence Microscopy Corrected by OPTiSPIM***

running title: ***Attenuation Artifacts in LSFM Corrected by OPTiSPIM***

Jürgen Mayer^1,2,3^, Alexandre Robert-Moreno^1,2,4^, James Sharpe^1,2,5,6^ & Jim Swoger^1,2,7^*

^1^ Centre for Genomic Regulation (CRG), The Barcelona Institute of Science and Technology, Dr. Aiguader 88, Barcelona 08003, Spain.

^2^ Universitat Pompeu Fabra (UPF), Barcelona, Spain.

^3^ Juergen.Mayer@bruker.com

^4^ alexandre.robert@embl.es

^5^ Institució Catalana de Recerca i Estudis Avançats (ICREA), Pg. Lluis Companys 23, Barcelona 08010, Spain.

^6^ james.sharpe@embl.es

^7^ jim.swoger@embl.es

* Corresponding author. Current address: European Molecular Biology Laboratory (EMBL) Barcelona, Dr. Aiguader 88, Barcelona 08003, Spain. e-mail: jim.swoger@embl.es. phone: +34 660 827 918.

**Table of Contents**

**Supplementary Text** Page 3

ATTENUATION CORRECTION IN A MURINE LYMPH NODE 3

ATTENUATION CORRECTION IN THE PRESENCE OF BACKGROUND / NOISE 4

SUPPLEMENTARY METHODS 6

Attenuation Correction Implementation 6

Sample Preparation 6

Scanning 7

Data Processing & Visualization 8

RESOLUTION & DEPTH OF FIELD CONSIDERATIONS IN OPT 10

**Supplementary Figures** 12

SUPPLEMENTARY FIGURES 1-6 12

**Supplementary Tables** 20

Supplementary Table 1: Sample Scan Parameters 20

Supplementary Table 2: Abbreviation & Symbol Definitions 21

**Supplementary References** 22

**Supplementary Text**

1. **Attenuation correction in a murine lymph node**.

A sample of interest to immunologists is the murine lymph node depicted in **Supplementary Fig. 3**. Unlike the embryonic mouse head, the lymph node has no intrinsic pigmentation, and therefore after optical clearing exhibits negligible attenuation. However in this case, in order to quantify the number and structure of B-cell follicles the lymph node was stained by *in situ* hybridization for the *Cxcl13* transcript, which leaves a non-fluorescent precipitate in cells that express the gene (see **Methods** (**Sample Preparation)** for details). **Supplementary Fig. 3a** shows a bright-field image of such a lymph node after dissection and *in situ* staining, but before optical clearing. The darker, purplish regions are caused by the nitro blue tetrazolium / 5-bromo-4-chloro-3-indolyl phosphate (NBT/BCIP) precipitate, which clearly indicate *Cxcl13* expression and hence the locations of the B-cell follicles. Our goal in imaging this sample was to use the tOPT mode of the OPTiSPIM to reconstruct the 3D distributions of the *Cxcl13* gene, and the SPIM mode to map the intrinsic autofluorescence of the lymph node tissue, which gives the overall geometry of the organ. As expected, the B-cell follicles reconstruct well (**Supplementary Fig. 3b**), but the autofluorescence signal is corrupted by shadow artifacts (**Supplementary Fig. 3c**). As with the embryonic mouse head, we use our 3D map of the attenuation (i.e. the absorbing stain in the follicles) to correct the fluorescence signal, which results in a data set from which most of the attenuation artifacts have been removed (**Supplementary Fig. 3d**). As a comparison, an optical section through an unstained lymph node is shown in **Supplementary Fig. 3e**; although the details of the organ structures are not expected to be the same, it can be clearly seen that the attenuation-corrected version (**Supplementary Fig. 3d**) is a better match to this control than the uncorrected image (**Supplementary Fig. 3c**). **Supplementary Fig. 3f** shows the intensity profiles along the lines indicated in **Supplementary Figs. 3c-e** in order to quantify this comparison. As an indication that this correction is quantitatively useful, isosurfaces of the uncorrected and corrected fluorescence data are shown in **Supplementary Figs. 3g** and **3h**, respectively. The presence of the attenuation artifacts in the uncorrected data clearly leaves large “pits” in the surface of the structure, and the lymph node volume calculated from this isosurface (0.183 mm^3^) significantly underestimates the actual volume (compare to the value of 0.231 mm^3^ for the corrected data set).

1. **Attenuation correction in the presence of background / noise**.

As mentioned in the **Discussion**, samples that include regions of very strong attenuation may result in the fluorescent emission being reduced to/below background levels. In such cases, the direct application of the Beer-Lambert law would result in amplification of the (unwanted) background/noise, rather than the (desired) fluorescent signal. **Supplementary Fig. 2** illustrates how our method avoids this pitfall by showing the effect of the background parameter *B* (see **Methods**) on the quality of the attenuation correction. A correct estimate of *B* yields an image (**Supplementary Fig. 2c**) that is a good approximation to the control image (**Supplementary Fig. 2b**). In contrast, when the background is ignored (*B* = 0, **Supplementary Fig. 2d**) in some regions (e.g. in the upper portion of the eye) the *AM* values are so low that the background/noise is amplified to levels much higher than the real fluorescence signal (see **eq. 16**).

Note that although *noise* in the image will be amplified by the attenuation correction process, the *signal-to-noise ratio* (SNR) is unaffected. This can be seen by considering the SNR before ($SNR_{raw}$) and after ($SNR_{cor}$) correction. Before correction, the signal is $F_{det}-B$ and the noise (or background) is $B$, so that $SNR_{raw}=\frac{F_{det}-B}{B}=\frac{F_{det}}{B}-1$. Using the notation of **eq. 16**, after correction the signal is $F_{est}$ and the noise $B\cdot\left[1+\frac{\left(F_{det}-B\right)\cdot\left(1-AM\right)}{AM\cdot F_{det}}\right]$. Using **eq. 16** and simplifying, the corrected signal-to-noise ratio is therefore $SNR_{cor}=\frac{F_{est}}{B\cdot\left[1+\frac{\left(F_{det}-B\right)\cdot\left(1-AM\right)}{AM\cdot F_{det}}\right]}=\frac{\left(F_{det}-B\right)\cdot\left[1+\frac{\left(F_{det}-B\right)\cdot\left(1-AM\right)}{AM\cdot F_{det}}\right]}{B\cdot\left[1+\frac{\left(F_{det}-B\right)\cdot\left(1-AM\right)}{AM\cdot F_{det}}\right]}=\frac{F_{det}}{B}-1$, so that $SNR_{cor}=SNR_{raw}$.

In regions where attenuation has reduced the desired signal to negligible levels, although our method does not amplify the background level, it also does not completely achieve our goal of removing attenuation artifacts in the fluorescence signal. Fortunately, the method we propose here is compatible with many of the attenuation-artifact-reduction techniques mentioned in the **Introduction**. Indeed, although they are not essential to our method we have used *chemical clearing**[[1]](#endnote-1)* and the light-sheet-tilting aspect of *mSPIM**[[2]](#endnote-2)* to generate the OPTiSPIM data we have present here, as these were obvious candidates to improve the quality of our images. Adding a second illumination arm as in mSPIM, or using *multi-photon fluorescence excitation* to reduce attenuation artifacts in the illumination process would also be straightforward to incorporate into our system design, although they would add somewhat to the complexity and cost.

On the conceptual level, the main difference between the computational post-processing approaches demonstrated in[[3]](#endnote-3),[[4]](#endnote-4) and our method (which also involves post-processing) is that they rely on assumptions about the statistical properties of the streak artifacts, whereas ours is based on experimental measurements of the sample’s attenuating properties. Nevertheless, their methods should at least in principle be compatible with ours, and we suggest that the methods of^3,4^ might be useful to remove the residual artifacts seen in our corrected images.

We expect that *multi-view image fusion* could be quite useful for reducing some residual artifacts that remain after our correction method has been applied, such as the small shadow that remains in **Fig. 4b**. However, multi-view fusion would be of little help in regions where the attenuation distribution is highly complex (see **Fig. 1a**), such as the retina inside the embryonic mouse eye bulb (**Supplementary Fig. 1a**)

Our method for correcting artifacts caused by attenuation of the illumination can be considered an alternative to the use of *self-healing beams* for LSFM illumination: since the illumination optically self-heals when attenuated, there will be less need for correction of attenuation artifacts. However, self-healing illumination beams do not reduce artifacts due to attenuation of the *detected* light, so we expect that our method could be used to good effect in combination with self-healing illumination by simply replacing our **eq. 7** with $AM=AM_{det}$ (see **Methods**).

1. **Supplementary Methods**.

**Attenuation Correction Implementation**

To numerically apply the above math to our measured data sets, and thus achieve our goal of correcting attenuation artifacts in them, we have written Matlab code (available on request) to

1. Reconstruct the attenuation coefficient map(s) $\alpha\left(\vec{r}\right)$ from the measured OPT projections, using a filtered back-projection,
2. Calculate the attenuation maps $AM_{ill}$ and $AM_{det}$ from the attenuation coefficient map(s) and the known parameters of our OPTiSPIM microscope, using **eqs. 17 & 18**, and
3. Invert the attenuation maps and use them to correct the fluorescence LSFM data, using **eqs. 7 & 16**.

**Sample Preparation**

**Murine lymph nodes** (LNs) were assessed for *Cxcl13* expression by in situ hybridization (ISH), a chromogenic technique in which the NBT and BCIP substrates are catalyzed to produce a colored precipitate, and therefore generate optical attenuation in the samples. We used popliteal LNs from 3 month-old mice; after dissection, lymph nodes were fixed overnight in 4% paraformaldehyde (Sigma) at 4°C, dehydrated into methanol using a methanol/PBS 0.1% tween-20 (PBT) series and left in methanol at -20°C until use. After rehydration through the same methanol/PBT series, lymph nodes were treated with 6% H_2_0_2_ (Sigma)/PBT for 1 hour at room temperature, digested with 10mg/ml of proteinase K for 20 minutes and refixed in 4% PFA, 0,2% glutaraldehyde for 40 minutes. Plasmid containing *Cxcl13* cDNA was used and digoxigenin-labelled *Cxcl13* riboprobe was synthesized following standard procedures. 0.5 to 2mg/ml of the probe was added to hybridization buffer and lymph nodes were incubated with the probe overnight rocking at 70°C. Following several washes of the probe at 70°C, samples were blocked for 90 minutes in TBS 0.1% tween-20/10% sheep serum, and then incubated with 1:2000 alkaline phosphatase coupled anti-digoxigenin antibody (Roche) in the same blocking solution overnight at 4°C. After extensive antibody washing in TBST with 2mM levamisole for 1 day at room temperature, lymph nodes were left in the same solution overnight at 4°C. LNs were washed three times for 10 minutes in NTMT (100mM NaCl, 100mM TrisHCl ph9.5, 50mM MgCl_2_, 0.1% tween) and developed with 45mg/ml NBT (Roche) and 175mg/ml BCIP (Roche) in NTMT solution. Note that all solutions were made in RNAse-free water by treating with DEPC (Sigma).

The **E12.5 embryonic mouse head** was dissected and fixed overnight in 4% paraformaldehyde at 4°C, blocked and permeabilized and incubated with anti-Tuj1 (for developing nerves) at 4°C. After extensive washes, samples were incubated with a secondary antibody coupled to Alexa488. The samples were then washed again and prepared for SPIM imaging.

To test our correction algorithm, **phantoms** with a simple geometry were produced: A small cylinder of 1% low gelling temperature agarose (Sigma, A9414-250G) containing diluted ink was embedded in a slightly larger cylinder of transparent agarose (see **Fig. 2c-j**). Both cylinders contain fluorescent beads in the same concentration. A liquid ink/beads/agarose mixture was drawn into a capillary with an inner diameter of 0.5 mm to form the small cylinder. Once solidified, the small cylinder was embedded in the larger one, which was then allowed to set. The larger cylinder (containing the smaller one) has a diameter of 1.1 mm.

**Embedding and clearing**. Subsequent to the processing described above, the embryonic mouse head and lymph node samples were mounted in 1% low melting agarose gel, dehydrated in methanol, and cleared in BABB (a 1:2 mixture of benzyl alcohol and benzyl benzoate) prior to imaging (see^1^ for details). The phantom samples were imaged in aqueous medium, without dehydration or clearing, because the fluorescent beads did not withstand chemical clearing in BABB.

The samples used in this work were selected as having relatively strong, well-localized absorbing regions because, in our experience, these are the most challenging. Samples with weak absorption with few sharp edges tend not to result in artifacts that are readily visible, and are thus of less concern to the experimenter than the strong “stripe” artifacts that our samples display. In addition, absorbing regions with discrete boundaries will induce stronger diffraction effects, which our method is not designed to correct. The fact that the images corrected by the method presented here demonstrate a significant reduction in attenuation-induced artifacts implies that samples with more diffuse absorption distributions will be similarly, or better, corrected.

**Scanning**

Detection utilized either a 4× objective lens (Leica, PLAN, NA = 0.1, WD = 26.2 mm) or a 5× objective (Leica, N PLAN EPI, NA = 0.12, WD = 14.0 mm) and a 12-bit cooled CCD camera with a pixel pitch of 6.45 µm (Hamamatsu ORCA-ER C4742-80). For single-sided SPIM illumination, a 2.5× air objective lens (Leica, NPLAN, NA = 0.07, WD = 11.2 mm) was used, with a 488 nm diode laser for fluorescence excitation. Note that although the objective lenses used were designed for working in air, because of their relatively low NAs we have not found significant aberrations when imaging up to several millimetres into aqueous or BABB-cleared samples. However, for larger samples (e.g. cleared adult mouse brains) or NAs aberrations are expected to become important, and switching to objectives designed for the appropriate immersion refractive index will be necessary for optimal results. For OPT transmission illumination, either a halogen reflector lamp or a 660 nm light-emitting diode was used. A diffuser guaranteed spatial incoherence of the tOPT illuminating light in both cases. When the halogen lamp was used as the tOPT illumination source, a filter was used in the detection light path to match the transmitted light spectrum as closely as possible to the corresponding fluorescence spectrum. See **Supplementary Fig. 4** and **Supplementary Table 1**.

Following the mSPIM model^2^, we used a resonant scan mirror (RSM) to tilt the light sheet around the centre of the camera’s field of view. In our system, the mirror operates at 1 kHz with a peak-to-peak oscillation amplitude of 15°. With our coupling optics this translates to a tilt angle range of the light sheet in the sample of 2.2°. Thus, when employing **eq. 17** we set and .

For tOPT imaging, we placed an iris in the back focal plane of the detection objective to reduce the NA and thereby control the imaging depth of field (DOF). The NA was reduced until the DOF was large enough to encompass the entire sample. Before acquiring the actual SPIM data used for our calculations, the iris was reopened so that the full resolution of the detection objective was obtained.

As the embryonic head shown in **Figs. 1 & 3** is larger than the field of view of the imaging system, the complete data set was collected by 3D tiling of the sample (lateral tile overlap 12%; vertical overlap 35%).

**Supplementary Table 1** summarizes the parameters for the scans of the individual samples.

**Data Processing & Visualization**

Numerical stitching of the 3D tiles generated by the SPIM scan of the embryonic mouse head was performed in FIJI[[5]](#endnote-5), using the “Grid/Collection Stitching” plug-in[[6]](#endnote-6). Because the data files for this sample are large (~16 Gbyte / channel) and the regions that cause attenuating artifacts (the eye bulbs) are localized, to save computational time attenuation correction was performed only on the sub-regions of the data in which attenuation was significant. Edge artifacts related to this can be observed above the eye in **Supplementary Fig. 2d** (in which the non-realistic assumption of zero background was used), although they are negligible when the background is properly accounted for in **Supplementary Fig. 2c**.

In principle the output from our OPTiSPIM apparatus includes the meta-data determining the spatial relationship between the OPT and SPIM images. However, larger data sets such as the embryonic mouse head require tiling of the SPIM data. Since this was done by spatially translating the component tiles in the “Grid/Collection Stitching” FIJI plug-in, the exact registration between different modalities can be lost. We therefore performed a “fine” registration by manually dithered the tOPT data sets by up to 3 voxels in each dimension, and determining the optimal registration by eye. By comparing the attenuation correction results for data with and without this fine registration, the end results were found to be registered to an accuracy of one voxel. For larger samples or those that require transformations more complex than simple translation for optimal tiling, more accurate and automated registration, e.g. using fiduciary markers such as beads, may be required.

For surface-rendered images used to compare uncorrected and corrected data (e.g. **Figs. 3b-c**, **Supplementary Fig. 1**, and **Supplementary Figs. 3g-h**), the same threshold level has been used for each of the image pairs that are compared. Similarly, grey-scale images to be compared (e.g. **Fig. 4** and **Supplementary Figs. 3c-e**) have been normalized so that the unattenuated regions have the same grey levels.

To enable visualization of dim sample features in the embryonic mouse head without saturating brighter ones, gamma adjustment of the fluorescence grey levels has been performed in **Fig. 1c,e** and **Fig. 4** (γ = 0.2), and **Supplementary Fig. 2** (γ = 0.5).

The images in **Fig. 2d,f,h,j** have been processed using the “Unsharp Mask” in FIJI^5^, with radius 1 pixel and mask weight 0.6, to enhance the visibility of the beads. See also **Supplementary Fig. 6**.

1. **resolution & Depth of field considerations in OPT**.

In OPT, successful 3D imaging requires that the images used in the reconstruction be well modelled by a mathematical projection through the sample. In practice this means that the depth of field (DOF) must be greater than the axial extent of the sample, so that the sample is entirely in focus as the projections are acquired. For an imaging system with a clear aperture, the (lateral) resolution ($\Delta x$) and DOF are given by[[7]](#endnote-7):

$\Delta x=\frac{0.61\lambda}{NA}$ **(S1)**

and

$DOF=\frac{n_{i}\cdot\lambda}{NA^{2}}$ **(S2)**

where $\lambda$ is the wavelength and $n_{i}$ is the refractive index of the imaging medium.

For simplicity, let us consider a sample that is roughly spherical, who’s size in all 3 dimensions is $\sim X$. For an accurate OPT reconstruction we require that in all images the sample be in focus; i.e. we need $X\leq DOF$. So to determine the maximum NA that can be used to properly image the sample we set $X=DOF$ in **eq. (S2)** and solve for the NA:

$NA_{\max}=\sqrt{\frac{n_{i}\cdot\lambda}{X}}$ **(S3)**

Substituting **eq. (S3)** into **(S1)** yields

$\Delta x_{\min}=\frac{0.61\lambda}{NA_{\max}}=\frac{0.61\lambda}{\sqrt{\frac{n_{i}\cdot\lambda}{X}}}=0.61\sqrt{\frac{X\cdot\lambda}{n_{i}}}$ , **(S4)**

which is the best resolution that OPT can achieve in a sample of size $X$ using a clear aperture.

Now consider the number of elements that it will be possible to resolve within this sample:

$N_{res}=\frac{X}{\Delta x_{\min}}$ **(S5)**

This gives a relative measure of how well the sample can be imaged: a large $N_{res}$ means that we can resolve components of the sample that are small relative to the sample size.

Substituting **eq. (S4)** into **(S5) we have**

$N_{res}=\frac{X}{0.61\sqrt{\frac{X\cdot\lambda}{n_{i}}}}=1.64\sqrt{\frac{n_{i}\cdot X}{\lambda}}$ **(S6)**

Equation **(S6)** tells us that $N_{res}\propto\sqrt{X}$, i.e. the smaller the sample size ($X$), the lower the relative resolution that can be achieved with OPT using a clear aperture. Thus when imaging small samples with OPT, to achieve a large $N_{res}$ it may be necessary to use techniques to extend the DOF (i.e. allow us to use an NA larger than that defined by **eq. (S3)**).

One technique that can be useful to extend the DOF is to take a 3D image stack (instead of a single 2D image) with a sufficiently high NA to achieve the desired resolution (according to **eq. (S1)**). The desired projection can then be computationally created *in silico*. This approach was applied by Fauver *et al*[[8]](#endnote-8) who, in order to achieve high resolution in single isolated cell nuclei, extended the DOF by making digital projections through 3D image stacks. Alternatively, PSF engineering techniques can be used to optically extend the DOF, e.g. by using an annular aperture to form a Bessel beam[[9]](#endnote-9).

**Supplementary Figures**


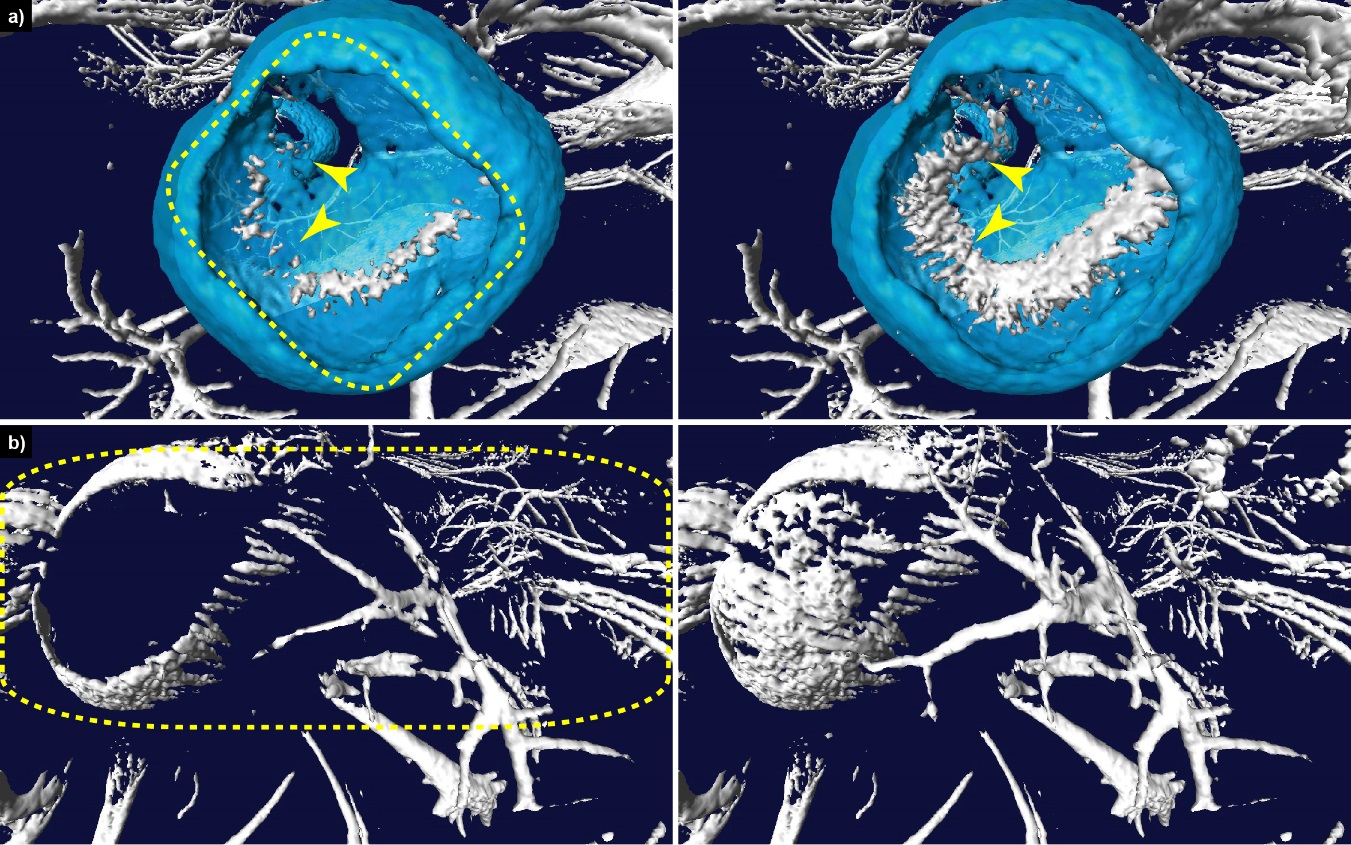


**Supplementary Figure 1.** Additional isosurface views of the embryonic mouse head from **Fig. 3**, highlighting different regions of the sample. Left: uncorrected; right: after attenuation correction. **a)** Correction of the fluorescence in the retina of the eye can be clearly seen. This is a region in which it would be problematic to achieve similar image quality using the mSPIM or multi-view imaging techniques alone, as the retina is almost entirely enclosed within the pigmented region of the eye. **b)** A region behind the eye bulb where correction is significant but incomplete, due to the high levels of attenuation that occur as the light sheet passes tangentially through the pigmented surface of the eye bulb. Yellow dashed shapes indicated regions where attenuation effects are significant; outside these regions, there are negligible attenuation artifacts. Yellow arrowheads indicate features that are absent in the uncorrected images, but are successfully recovered after correction.


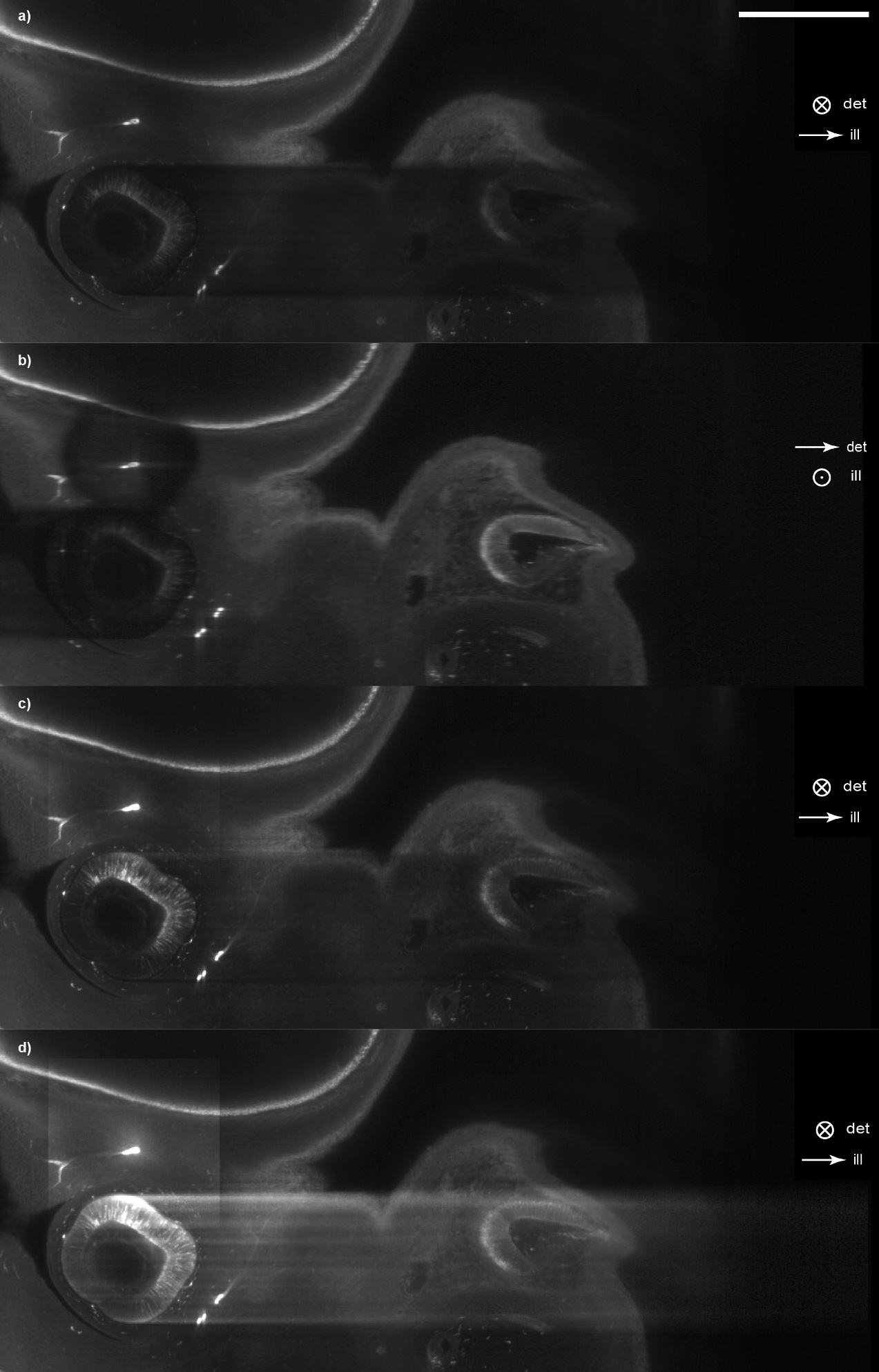


**Supplementary Figure 2.** Attenuation correction in the embryonic mouse head, showing overcorrection when background/noise is ignored. The sample is the same one as in **Figs. 1 & 3**. **a)** uncorrected. **b)** uncorrected control (scanned after 90° rotation about the vertical axis as in **Fig. 4c**, but note the different viewing orientation). The region to the right of the eyes is largely unattenuated, and can be used as a reference. **c)** data set from **a)**, corrected with *B* = 500 (best estimate of background level, **eq. 16**). **d)** data set from **a)**, corrected with *B* = 0 (assumption of no background, where **eq. 16** reduces to **eq. 9**). Some of the grey levels in **c)** & **d)** have been allowed to saturate, in order to visualize the dimmer regions.


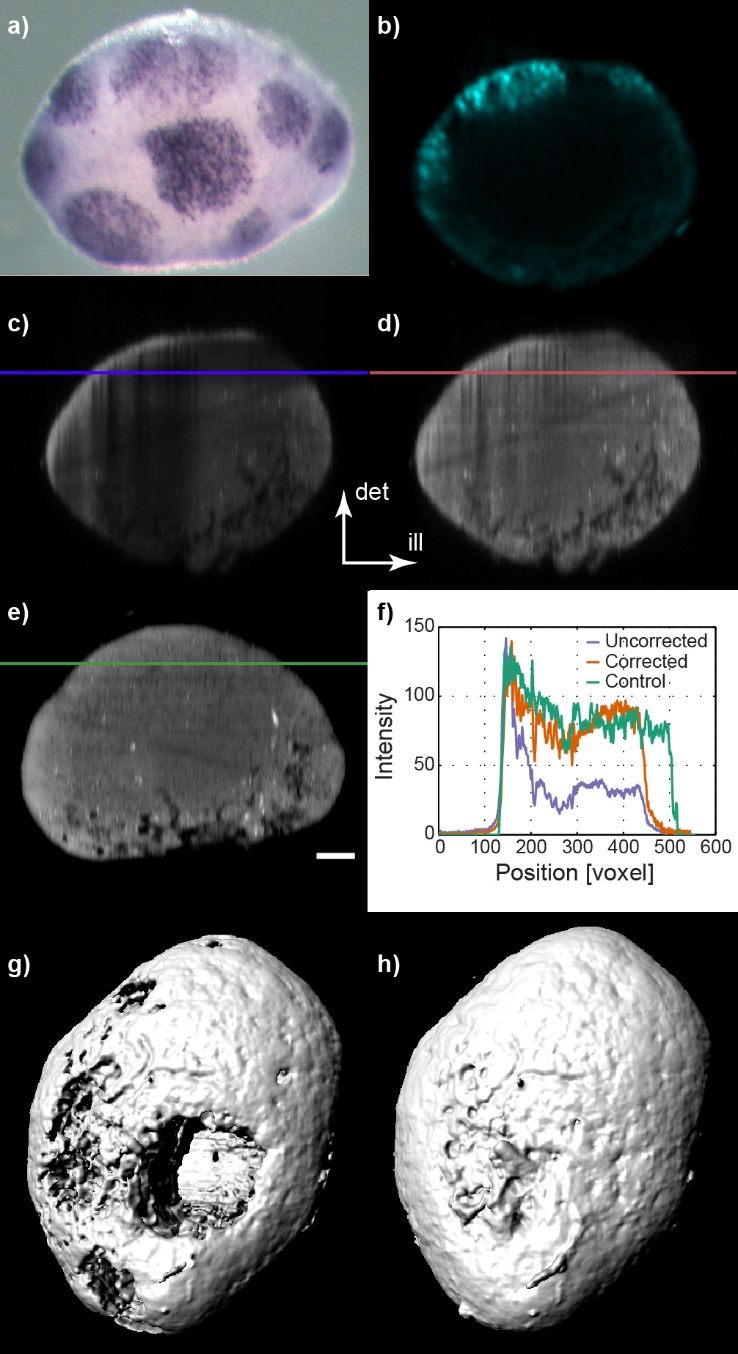


**Supplementary Figure 3.** Attenuation correction in an *in situ* hybridization (ISH) stained murine lymph node. **a)** Brightfield image of an uncleared lymph node stained by ISH with NBT/BCIP. **b)** Attenuation map (colors inverted for better visibility). **c)** Recorded fluorescent SPIM signal. **d)** Corrected fluorescent SPIM signal. **e)** Control LN without ISH and therefore no precipitation of NBT/BCIP. **f)** Intensity profiles of the lines in (**c, d, e**): recorded fluorescence signal (violet), corrected fluorescence signal (orange), fluorescent signal from control LN (green). **g-h)** Surface renderings of the lymph node before and after correction of the fluorescent SPIM signal. **g)** Before correction attenuation of the fluorescent signal leads to an underestimation of the LN volume (~0.183 mm^3^), visible as “pits” in the surface rendering. **h)** After application of the attenuation correction the surface approximates the natural shape of a LN much better (volume ~0.231 mm^3^). Scale bars 100 µm.


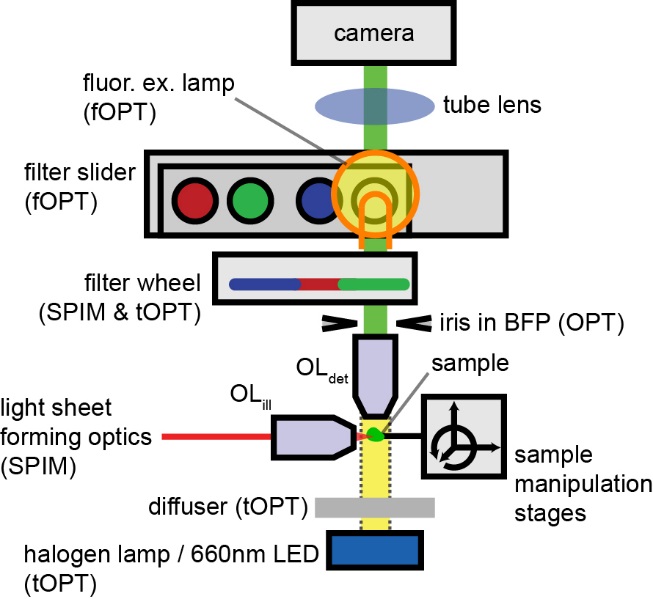


**Supplementary Figure 4.** The OPTiSPIM scheme (top view): The laser beam for the light sheet comes in from the left; detection is towards the top; transmission illumination (for tOPT) from the bottom. The filter wheel is used for SPIM & tOPT modes; the filter slider and fluorescence excitation lamp are for fluorescence OPT (fOPT, not used in the present work). The sample immersion chamber is not shown. OL_ill_: illumination objective lens, OL_det_: detection objective lens, BFP: back focal plane.


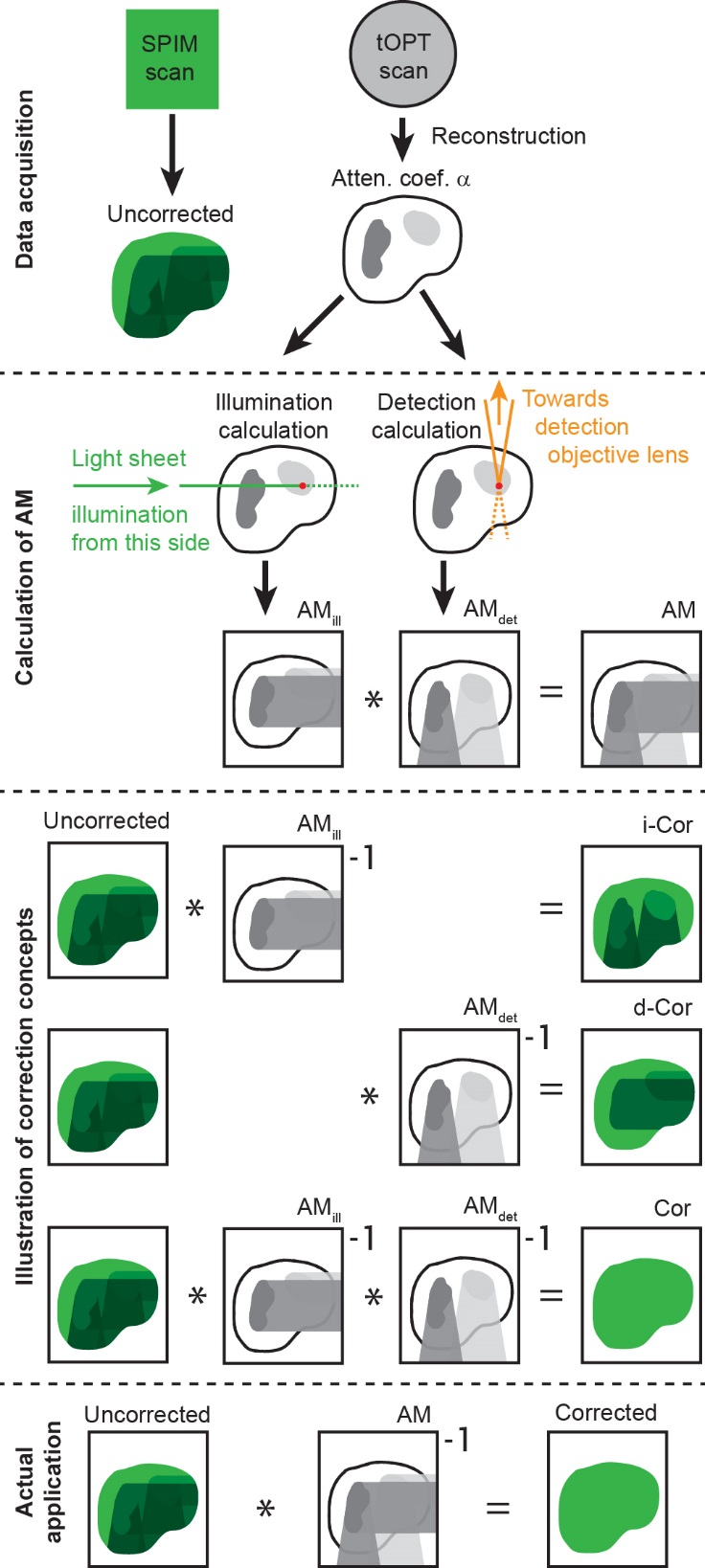


**Supplementary Figure 5.** Overview over the attenuation correction calculation process: A fluorescent SPIM scan provides the uncorrected fluorescent 3D data. The different views of the transmission OPT (tOPT) scan are reconstructed to deliver the 3D distribution of the attenuation coefficient (α) of the sample. The same α is used for the calculation of both the illumination (*AM_ill_*) and detection (*AM_det_*) attenuation maps. The system attenuation map (*AM*) is simply the product of *AM_ill_* and *AM_det_*. The uncorrected SPIM data is multiplied by the inverse of *AM* to calculate the corrected fluorescent signal (Cor). If only (*AM_ill_*) is applied, we get illumination corrected data (i-Cor); if only (*AM_det_*) is applied, we get detection corrected data (d-Cor).

**
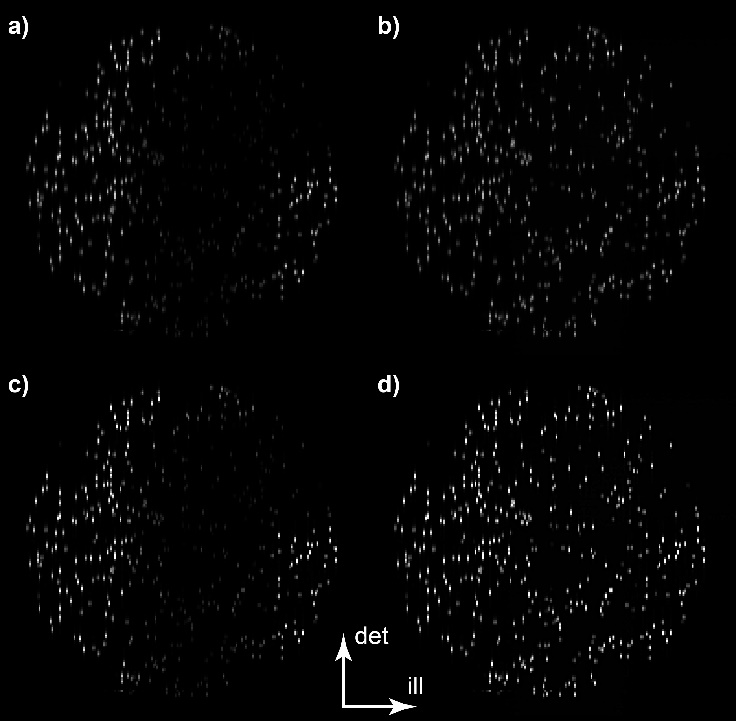
**

**Supplementary Figure 6.** Comparison of raw bead images (a-b, without Unsharp Mask filtering) and with Unsharp Mask filtering (c-d). c) and d) are the same images as are shown in **Fig. 2d** and **j**, respectively. The Unsharp Mask was used purely to make the beads more visible in the figure; it does not contribute to the attenuation correction procedure.

**Supplementary Tables**

| **Parameter** | **Embryonic Mouse Head** | **Lymph Nodes** | **Bead Phantom** |
| --- | --- | --- | --- |
| *K_ill_* | 4 | 1 | 1 |
| *K_det_* | 10 | 1 | 1 |
| *B* ^#^ | 500 | 2 | 1 |
| SPIM excitation λ | 488 nm | 488 nm | 488 nm |
| SPIM emission λ | 500-550 nm | 500-550 nm | 500-550 nm |
| SPIM slice spacing | 5 µm | 5 µm | 5 µm |
| tOPT source | 660 nm LED | halogen lamp | halogen lamp |
| tOPT filter_ex_ λ ^*^ | none | 470-510 nm | 470-510 nm |
| tOPT filter_em_ λ ^**^ | none | 500-550 nm | 500-550 nm |
| tOPT angular increment | 0.9° | 0.9° | 0.9° |
| illumination objective | 2.5× / 0.07 | 2.5× / 0.07 | 2.5× / 0.07 |
| detection objective | 5× / 0.12 | 4× / 0.1 | 4× / 0.1 |

**Supplementary Table 1: Sample Scan Parameters**

^#^ Note that for technical reasons background subtraction was done before the attenuation correction processing for the lymph node and phantom samples, but not for the embryonic mouse head.

^*^ approximates the SPIM fluorescence excitation spectra

^**^ approximates the SPIM fluorescence emission spectra

| **Abbreviation / Symbol** | **Interpretation** |
| --- | --- |
| *α* | optical attenuation coefficient |
| *AM* | attenuation map |
| *AM_det_* | detection attenuation map |
| *AM_ill_* | illumination attenuation map |
| *B* | fluorescence background level |
| BABB | 1:2 mixture of benzyl alcohol and benzyl benzoate |
| BCIP | 5-Bromo-4-chloro-3-indolyl phosphate |
| DOF | depth of field |
|  | minimum & maximum scan angles for the RSM |
| *F*_0_ | ‘real’ fluorescence signal |
| *F_det_* | measured fluorescence signal |
| *F_est_* | our best estimate of *F*_0_ |
| fOPT | fluorescence OPT |
| ISH | *in situ* hybridization |
| *K_det_* | ratio of $\alpha(\lambda_{\det}):\alpha(\lambda_{measured})$ |
| *K_ill_* | ratio of $\alpha(\lambda_{ill}):\alpha(\lambda_{measured})$ |
| $\lambda_{det}$ | detection wavelength |
| $\lambda_{ill}$ | illumination wavelength |
| LN | lymph node |
| LSFM | light sheet fluorescence microscopy |
| mSPIM | multi-directional SPIM |
| MRI | magnetic resonance imaging |
| NA | numerical aperture |
| NBT | nitro blue tetrazolium |
| *n_i_* | refractive index |
| OPT | optical projection tomography |
| OPTiSPIM | a hybrid platform combining OPT & SPIM |
| RSM | resonant scan mirror |
| *S* | fluorescence correction weighting factor |
| SPIM | selective plane illumination microscopy |
| $\vartheta_{\max}$ | objective lens acceptance angle |
| tOPT | transmission OPT |
| WD | working distance |

**Supplementary Table 2: Abbreviation & Symbol Definitions**

**References**

1. Sharpe, J. *et al*. “Optical Projection Tomography as a Tool for 3D Microscopy and Gene Expression Studies” *Science* **296**, 541-545 (2002). [↑](#endnote-ref-1)
2. Huisken, J. & Stainier, D.Y.R. “Even fluorescence excitation by multidirectional selective plane illumination microscopy (mSPIM)” *Optics Letts.* **32**, 2608-2610 (2007). [↑](#endnote-ref-2)
3. Fehrenbach, J., Weiss, P. & Lorenzo, C. “Variational Algorithms to Remove Stationary Noise: Applications to Microscopy Imaging” *IEEE Trans. Image Process.*, **21**(10), 4420-4430 (2012). [↑](#endnote-ref-3)
4. Leischner, U., Schierloh, A., Zieglgänsberger, W. & Dodt, H.U. “Formalin induced fluorescence reveals cell shape and morphology in biological tissue samples” *PLoS One* **5**, e10391 (2010). [↑](#endnote-ref-4)
5. Schindelin, J. *et al*. “Fiji - an open source platform for biological image analysis” *Nat. Methods* **9**(7), 676-682 (2012). [↑](#endnote-ref-5)
6. Preibisch, S. *et al*. “Globally Optimal Stitching of Tiled 3D Microscopic Image Acquisitions” *Bioinformatics* **25**(11), 1463-1465 (2009). [↑](#endnote-ref-6)
7. “Handbook of Biological Confocal Microscopy, 3^rd^ Ed.” Pawley, J. editor. Springer Science+Business Media LLC, New York NY USA (2006). [↑](#endnote-ref-7)
8. Fauver, M. *et al*. “Three-dimensional imaging of single isolated cell nuclei using optical projection tomography” *Optics Express* **13**(11), 4210-4223 (2005). [↑](#endnote-ref-8)
9. Linfoot, E.H. & Wolf, E. “Diffraction Images in Systems with an Annular Aperture” *Proc. Phys. Soc. B* **66**(2) 145-149 (1953). [↑](#endnote-ref-9)
